# Supplementary material for: Entropy based analysis of vertebrate sperm protamines sequences: evidence of potential dityrosine and cysteine-tyrosine cross-linking in sperm protamines
Source: BMC Genomics. 2020 Apr 3;21:277. doi: 10.1186/s12864-020-6681-2 (PMC7126135; doi:10.1186/s12864-020-6681-2)
Supplement: Supplementary file 1 — Additional file 1 The additional figures and tables referenced within this article are available in the figshare repository, https://doi.org/10.6084/m9.figshare.10292573.v1 [40]. The supplemental information file (.pdf) can be found in the.zip archive under ProtamineAnalysisFigshare/Docs/supplemmental.pdf. [file 12864_2020_6681_MOESM1_ESM.pdf]

Entropy Based Analysis of Vertebrate Sperm Protamines Sequences: Evidence of Dityrosine and Cysteine-Tyrosine Cross-Linking in Sperm Protamines

Supplemental Tables

Table 1 Highly Conserved Positions in Eutherian P1 Sperm Protamine MSA.

Rank	Position in Alignment	Relative Entropy (bit)	Represented Residues
1	7	6.157	C: 98.6%<br>R: 1.4%
2	49	6.12	C: 97.9%<br>G: 1.4%<br>S: 0.7%
3	50	5.87	C: 95.1%<br>R: 2.1%<br>Y: 1.4%<br>G: 0.7%<br>S: 0.7%
4	60	5.439	C: 89.5%<br>R: 4.9%<br>S: 4.2%<br>Y: 1.4%
5	38	5.187	C: 86.9%<br>G: 4.1%<br>Y: 2.8%<br>R: 1.4%<br>S: 1.4%<br>V: 1.4%<br>A: 0.7%<br>F: 0.7%<br>H: 0.7%
6	17	4.885	C: 79.3%<br>R: 13.1%<br>G: 5.5%<br>S: 1.4%<br>Y: 0.7%
7	29	4.813	C: 77.2%<br>Y: 13.1%<br>S: 5.5%<br>G: 1.4%<br>R: 1.4%<br>F: 0.7%<br>N: 0.7%
8	6	4.604	C: 95.5%<br>R: 4.5%
9	37	4.33	C: 69.0%<br>S: 19.3%<br>F: 5.5%<br>R: 5.5%<br>T: 0.7%

Table 2 Highly Conserved Positions in Processed Eutherian P2 Sperm Protamine MSA.

Rank	Position in Alignment	Relative Entropy (bit)	Represented Residues
1	59	6.381	C: 100.0%
2	75	6.381	C: 100.0%
3	83	6.381	C: 100.0%
4	93	6.381	C: 100.0%
5	107	6.381	C: 100.0%
6	68	5.513	H: 100.0%
7	89	4.721	H: 87.5%<br>N: 6.2%<br>P: 6.2%
8	53	4.635	H: 87.5%<br>G: 6.2%<br>S: 6.2%
9	85	4.543	H: 81.2%<br>Y: 12.5%<br>Q: 6.2%
10	110	4.371	H: 75.0%<br>Y: 18.8%<br>Q: 6.2%
11	54	4.288	Y: 56.2%<br>H: 43.8%

Table 3 Highly Conserved Positions in Unprocessed Eutherian P2 Sperm Protamine MSA.

Rank	Position in Alignment	Relative Entropy (bit)	Represented Residues
1	59	6.381	C: 100.0%
2	75	6.381	C: 100.0%
3	83	6.381	C: 100.0%
4	93	6.381	C: 100.0%
5	107	6.381	C: 100.0%
6	14	5.513	H: 100.0%
7	68	5.513	H: 100.0%
8	48	5.126	H: 93.8%<br>Q: 6.2%
9	44	5.093	Y: 100.0%
10	4	4.836	Y: 93.8%<br>C: 6.2%
11	19	4.725	Q: 100.0%
12	26	4.725	Q: 100.0%
13	34	4.725	Q: 100.0%
14	89	4.721	H: 87.5%<br>N: 6.2%<br>P: 6.2%
15	53	4.635	H: 87.5%<br>G: 6.2%<br>S: 6.2%
16	85	4.543	H: 81.2%<br>Y: 12.5%<br>Q: 6.2%
17	110	4.371	H: 75.0%<br>Y: 18.8%<br>Q: 6.2%
18	54	4.288	Y: 56.2%<br>H: 43.8%
19	47	4.171	T: 100.0%

Table 4 Highly Conserved Positions in Metatherian P1 Sperm Protamine MSA.

Rank	Position in Alignment	Relative Entropy (bit)	Represented Residues
1	7	4.98	H: 92.6%<br>R: 5.3%<br>N: 2.1%
2	4	4.835	Y: 95.8%<br>C: 3.2%<br>S: 1.1%
3	57	4.733	Y: 93.7%<br>C: 4.2%<br>K: 2.1%
4	16	4.67	Y: 94.7%<br>N: 3.2%<br>F: 2.1%
5	62	4.599	Y: 92.6%<br>C: 3.2%<br>P: 2.1%<br>S: 2.1%
6	75	4.565	Y: 94.5%<br>C: 3.3%<br>N: 2.2%
7	34	4.536	Y: 91.6%<br>S: 4.2%<br>Q: 2.1%<br>M: 1.1%<br>X: 1.1%

Table 5 Fish Protamines

Rank	Range		STD	
1	K	0.141	K	0.027
2	H	0.185	H	0.035
3	L	0.222	L	0.039
4	T	0.228	T	0.041
5	A	0.256	E	0.045

Table 6 Eutherian P1 Sperm Protamines

Rank	Range		STD	
1	Q	0.182	Q	0.034
2	K	0.224	P	0.044
3	T	0.224	T	0.046
4	S	0.242	N	0.046
5	N	0.244	A	0.049

Table 7 Eutherian P2 Sperm Protamines

Rank	Range		STD	
1	Q	0.07	Q	0.017
2	L	0.09	T	0.022
3	T	0.09	L	0.023
4	K	0.09	E	0.024
5	C	0.099	V	0.025

Table 8 Truncated Eutherian P2 Sperm Protamines

Rank	Range		STD	
1	Q	0.116	K	0.035
2	K	0.117	Q	0.037
3	T	0.127	T	0.04
4	L	0.145	L	0.044
5	C	0.151	G	0.045

Table 9 Metatherian Sperm Protamines

Rank	Range		STD	
1	K	0.177	N	0.027
2	N	0.179	K	0.03
3	G	0.202	T	0.035
4	A	0.223	A	0.035
5	E	0.235	Q	0.035

```
                           10         20         30         40
PRTA_ACIST       -ARRRRRHAS TKLKRRR--- -----RRRRH GKKSHK
PRT_ORYLA        ----MRRQAS LPARRRRRVR RTRVVRRRRR VGRRRH
PRT_PERFV        --PRRRRHAA RPVRRRRRTR RSSRVHRRRR AVRRRR
PRTB_MUGCE       --PRRRRETS RPIRRRRRAR RAPI-RRRRR VVRRRR
PRT1_SCOSC       -MPRRRRRAS RPVRRRRRAR RSTAVRRRRR VVRRRR
PRT2_SCOSC       -MPRRRRRAS RPIRRRRRAR RSTAVRRRRR VVRRRR
PRT_DICLA        --PRRRRQAS RPVRRRRRTR RSTAERRRRR VVRRRR
PRTY_THUTH       --PRRRRQAS RPVRRRRRYR RSTAARRRRR VVRRRR
PRTZ_THUTH       --PRRRRRSS RPVRRRRRYR RSTVARRRRR VVRRRR
PRTZ1_SAROR      --PRRRRRSS RPVRRRRRYR RSTAARRRRR VVRRRR
PRTB_ACIGU       --ARRRRRSS RPQRRRRR-- -----RRHGR RRRGRR
PRTB_ACIST       --ARRRRRSS RPQRRRRR-- -----RRHGR RRRGRR
PRTY1_CLUHA      --ARRRRSSS RPIRRRRPRR RT---TRRRR AGRRRR
PRTY1_CLUPA      --ARRRRSSS RPIRRRRPRR RT---TRRRR AGRRRR
PRT1_ESOLU       -PRRRRASSG RPVRRRRRP- KMS--RRRRR GGRRRR
PRTZ_CLUPA       ARRRRSRRAS RPVRRRRPR- RVS--RRRR- -ARRRR
PRTZ_CLUHA       ARRRRSRRAS RPVRRRRPR- RVS--RRRR- -ARRRR
PRT4_ONCMY       -MPRRRR-AS RRIRRRRRP- RVS--RRRRG GRRRRR
PRTY2_CLUPA      -PRRRTRRAS RPVRRRRPR- RVS--RRRR- -ARRRR
PRTY2_CLUHA      -PRRRTRRAS RPVRRRRPR- RVS--RRRR- -ARRRR
PRT3A_ONCMY      -PRRRRRSSS RPIRRRRRP- RVS--RRRRR GGRRRR
PRTIB_ONCMY      PRRRRRRSSS RPIRRRRPR- RVS--RRRRR GGRRRR
PRT1B_ONCMY      -MPRRRR-AS RRIRRRRRP- RVS--RRRRR GGRRRR
PRT1A_ONCMY      -MPRRRR-AS RRVRRRRRP- RVS--RRRRR GGRRRR
PRTC3_ONCMY      -MPRRRR-AS RPVRRRRRP- RVS--RRRRR GGRRRR
PRT2C_ONCMY      -MPRRRRSSR RPVRRRRRP- RVSR-RRRRR GGRRRR
PRT14_ONCMY      -MPRRRRSSR PPVRRRRRP- RVSR-RRRRR GGRRRR
PRT2_ONCMY       --PRRRRSSS RPVRRRRAR- RVSR-RRRRR GGRRRR
PRT2A_ONCMY      --PRRRRSSS RPVRRRRRA- RVSR-RRRRR GGRRRR
PRTIA_ONCMY      --PRRRRSSS RPVRRRRRPR RVSR-RRRRR GGRRRR
PRT1_ONCKE       -MPRRRRSSS RPVRRRRRP- RVSR-RRRRR GGRRRR
PRT2B_ONCMY      -MPRRRRSSS RPVRRRRRP- RVSR-RRRRR GGRRRR
PRT16_ONCMY      -MPRRRRSSS RPVRRRRRA- RVSR-RRRRR GRRRRR
PRT17_ONCMY      -MPRRRRSSS RPVRRRRRP- RVSR-RRRRR GRRRRR
```

Supplemental Figure 1. Alignment of fish protamines.

```
                           10         20         30         40         50         60
A0A2K5CXY9_AOTNA MARHRCCRSR --SQSRSR-R --DRQKRRCR -TPRRRSCRR --RTA-RRCG RRR--YKPRC RRN-
HSP1_RHIFE       MARYSCCRSH --SRSRSR-R ---R-RQRCR -RRRRRSCGR --RR--RACY RRYTVRYRRR RRRR
HSP1_HIPCO       MARYRCCRSH --SRSRCR-R ---R-RRRSR -RRRRRSCGR --RR--RAGY RRY---TVRY RRRR
A8IYA4_ANTAM     MARYRCCLTH --SRSRCRPR ---RRRRRCR -KLRRRFCRR -PRR--RVCC RRY--TAIRC TR--
A8IYA7_9CETA     MARYRCRLTH --SRSGCR-R ---RRRRRCR -RRRRRFCRR --RRR-RVCC RRY--TVVRC TRQ-
HSP1_CAPHI       MARYRCCLTH --SRSRCR-R ---RRRRRCR -RRRRRFGRR --RRR-RVCC RRY--TVVRC TRQ-
HSP1_SHEEP       MARYRCCLTH --SRSRCR-R ---RRRRRCR -RRRRRFGRR --RRR-RVCC RRY--TVVRC TRQ-
A8IYA2_OVIDA     MARYRCCLTH --SRSRCR-R ---RRRRRCR -RRRRRFGRR --RRR-RVCC RRY--TVVRC TRQ-
HSP1_BOVIN       MARYRCCLTH --SGSRCR-R ---RRRRRCR -RRRRRFGRR --RRR-RVCC RRY--TVIRC TRQ-
A0A068B2A1_BOSIN MARYRCCLTH --SGSRCR-R ---RRRRRCR -RRRRRFGRR --RRR-RVCC RRY--TVIRC TRQ-
A0A193KZW0_9CETA MARYRCCLTH --SGSRCR-R ---RRRRRCR -RRRRRFGRR --RR------ -RR------- ----
C8C436_PLAMN     MARNRCCRSQ --SRSRCR-R ---P-KRGCR -SRRRRCYQR --RR--RVCC RRY--TTIRC ARQ-
A8IYB4_PHYMC     MARNRCCRSQ --SRSRCR-R ---P-RRRCR -SPRRRRYQR --RR--RVCC RRY--TVTRC ARQ-
C8C437_KOGSI     MARNRCCRSQ --SRGRCR-R ---P-RRRCR -SPKRRRYQR --RR--RVCC RRS--ATMRC ASQ-
C8C438_KOGBR     MARNRCCRSQ --SRGRCR-R ---P-RRRYR -SPRRRRYQR --RR--RVCC RRS--TTMRC ASQ-
HSP1_PIG         MARYRCCRSH --SRSRCR-P ---R-RRRCR -RRRRRCCPR --RRR-AVCC RRY--TVIRC RRC-
A8IYC1_POTPR     MARYRCCRSH --SRSRCR-P ---R-RRRCR -RRRRRCCPR --RRR-AVCC RRY--TVIRC RRC-
F7VJK3_FELCA     MARYRCCRSH --SRSRCR-R ---R-RRRCR -RRRRRCCRR -PRK--RVCS RRY--RVGRC RRR-
HSP1_OTOHE       MARYRCCRSR --SRSRCR-R ---R-RRKCY -RRRRRCSRK --RRR-RVCC RRY--TVMRC RRR-
C8C444_HEXLI     MARYRCCRSP --SRSRCR-R ---Q-RRRCR -RRRRRCCRQ --RRR-RVCC RRY--TMVRC TRQ-
A8IYB9_HIPAM     MARYRCCRSP --SRSRCR-R ---Q-RRRCR -RRRRRCCRQ --RRR-RVCC RRY--TMVRC TRQ-
HSP1_EQUAS       MARYRCCRSQ --SQSRCR-R ---RRRRRCR -RRRRRCVRR --RR---VCC RRY--TVLRC RRRR
HSP1_HORSE       MARYRCCRSQ --SQSRCR-R ---RRRRRCR -RRRRRSVRQ --RR---VCC RRY--TVLRC RRRR
HSP1_HYPSA       MARYRRCR-- --SRSRCR-R ---R-RRRCH -RRRRRCCRR --RRRRRACC RRY-----RC RRR-
HSP1_NEOBU       MARYRRCR-- --SRSRCR-R ---R-RRRCH -RRRRRCCRR --RRRRRACC RRY-----RC RRR-
HSP1_RHIHA       MARYRCCRSR --SRSRCRPR -----RRRCR -RRRRRCCRR --RR--RVCC RRY---SARC RRRR
HSP1_MURCY       MARYRCCR-- --SRSRCR-R ---R-RRRCH -RRRRRCSRR --RRR-RVCC RRY--TVIRC RRR-
HSP1_PTEPA       MARYRCCRSP --SRSRCR-R ---R-RRRCR -RRRRRCCRR --RR--RVCC RRY---TVRC RRR-
HSP1_DESRO       MARYRCCRSP --SRSRCR-R ---R-RRRCR -RRRRRCCRR --RR--RVCC RRY---TVRC RRR-
HSP1_MONRE       MARYRCCRSP --SRSRCR-R ---R-RRRCR -RRRRRCCRR --RR--RVCC RRY---TVRC RRR-
HSP1_MORME       MARYRCCRSP --SRSRCR-R ---R-RRRCR -RRRRRSCRR --RR--RVCC RRY---TVRC RRR-
HSP1_MYODA       MARYRCCR-- --SRSRCR-R ---R-RRRCY -RRRRRCCRR --RRRRRVCC RRY----SRC RRR-
F7VJK7_MYOLU     MARYRCCR-- --SRSRCR-R ---R-RRRCY -RRRRRCCRR --RRRRRVCC RRY----SRC RRR-
HSP1_GLABE       MARYRCCR-- --SRSRCR-R ---R-RRRSY -RRRRRCCRR --RRR-RVCC RRY----VRC RRR-
HSP1_NATST       MARYRCCRSQ --SRSRCR-P ---R-RRRCR -TRRRRCCRR -RRR--RVCC RRY--TVVRC RRR-
HSP1_CHIMC       MARYRCCRSQ --SRSRCR-R ---R-RRRCR -TRRRRCCRR -RRR--RVCC RRY--TVVRC RRR-
HSP1_EPTFU       MARYRCCR-- --SRSRCR-R ---R-RRRCY -RRRRRCCRR -RRR--RVCC RRY--TVIRC RRR-
HSP1_EPTBR       MARYRCCR-- --SRSRCR-R ---R-RRRCY -RRRRRCCRR -RRR--RVCC RRY--TVIRC RRR-
HSP1_CORTO       MARYRCCRSQ --SRSRCR-R ---R-RRRCY -RRRRRCCRR --RRR-RVCC RRY----TRY RRR-
HSP1_PLEAU       MARYRCCRSQ --SRSRCR-R ---R-RRRCY -RRRRRCCRR -RRR--RVCC RRY--TVVRC RRR-
HSP1_PTEHP       MARYRCCRSQ --SRSRCR-R ---R-RRRCR -RRRRRCCRR --RR--RVCC RRY---TVRC RRRR
F7VJK4_CANLF     MARYRCCRSQ --SRSRCR-R ---R-RRRCR -RRRRRCCRR RRR---RVCC RRY--TVVRC RRR-
C8C440_EUBGL     MARNRCCRSQ --SRSRRR-R ---P-RQRCR -SQRRRCCRR --RR--RVCC RRY--TTVRC ARR-
C8C439_EUBAS     MARNRCCRSQ --SRSRRR-R ---P-RQRCR -SQRRRCCRR --RR--RVCC RRY--TTVRC ARR-
B1ACJ7_BALMY     MARNRCCRSQ --SRSRRR-R ---P-RQRCR -SQRRRCCRR --RR--RVCC RRY--TTVRC ARR-
B1ACJ8_EUBJA     MARNRCCRSQ --SRSRRR-R ---P-RQRCR -SQRRRCCRR --RR--RVCC RRY--TTVRC ARR-
B1ACJ6_CAPMR     MARNRCCRSQ --SRSRRR-R ---P-RQRCR -SQRRRCCRR --RR--RVCC RRY--TTVRC ARQ-
B1ACI8_MEGNO     MARNSCCRSP --SRSRRR-R ---P-RQRRR -SQRRRCCQR --RR--RVCC RRY--TTVRC ARQ-
B1ACI9_BALPH     MARNSCCRSP --SRSRRR-R ---P-RQRRR -SQRRRCCQR --RR--RVCC RRY--TTVRC ARQ-
B1ACJ3_BALBN     MARNSCCRSP --SRSRRR-R ---P-RQRCR -SQRRRCCRR --RR--RVCC RRY--TTVRC AGQ-
B1ACJ4_BALAC     MARNSCCRSP --SRSRRR-R ---P-RQRCR -SQRRRCCRR --RR--RVCC RRY--TTVRC AGQ-
B1ACJ1_BALBO     MARNSCCRSP --SRSRRR-R ---P-RQRCR -SQRRRCCRR --RR--RVCC RRY--TTVRC ARQ-
B1ACJ5_ESCRO     MARNSCCRSP --SRSRRR-R ---P-RQRCR -SQRRRCCRR --RR--RVCC RRY--TTVRC ARQ-
B1ACJ2_BALED     MARNSCCRSP --SRSRRR-R ---P-RQRCR -SQRRRCCRR --RR--RVCC RRY--TTVRC ARQ-
B1ACJ0_BALMU     MARNTCCRSP --SRSRRR-R ---P-RQRCR -SQRRRCCRR --RR--RVCC RRY--TTVRC ARQ-
C8C435_BERBI     MARNRCCRSQ --SQSRRR-R ---P-RRRNR -SRRRQCCQR --RR--RVCC RRY--TAIRC ARQ-
A8IYB7_ZIPCA     MARNRCCRGQ --SQSRRR-R ---P-RRRYR -SRRRQCCQK --RR--RVCC RRY--TATRC ARQ-
C8C432_MESGR     MARNTCCRSQ --SQSRRR-R ---P-RRRYR -SRRKQCCQK --RR--RVCC RRY--TAIRC ARQ-
C8C434_TASSH     MARNRCCRSQ --SQSRRR-R ---P-RRRYR -SRRRQCCQK --RR--RVCC RRY--TAIRC ARQ-
C8C433_MESBI     MARNRCCRSQ --SQSRRR-R ---P-RRRYR -SRRRQCCQK --RR--RVCC RRY--TAIRC ARQ-
C8C431_MESPE     MARNRCCRSQ --SQSRRR-R ---P-RRRYR -SRRKQCCQK --RR--RVCC RRY--TAIRC ARQ-
HSP1_ORCOR       MARNR-CRSP --SQSRCR-R ---P-RRRCR --RRIRCCRR --QR--RVCC RRY--TTTRC ARQ-
C8C425_GLOMA     MARNR-CRSP --SQSRCR-R ---P-RRRYR --RRRQCCRR --RR--RVCC RRY--TTTRC ARQ-
C8C426_GRAGR     MARNR-CRSP --SQSRCR-R ---P-RRRYR --RRRQCCRR --RR--RVCC RRY--TTTRC ARQ-
B4YVM8_PSECS     MARNR-CRSP --SQSRCR-R ---P-RRRYR --RRRQCCRR --RR--RVCC RRY--TTTRC ARQ-
C8C424_FERAT     MARNR-CRSP --SQSRCR-R ---P-RRRYR --RRRRCCRR --RR--RVCC RRY--TTTRC ARQ-
F5CBD0_PENEL     MARNR-CRSP --SQSRCR-R ---P-RRRYR --RRRRCCRR --RR--RVCC RRY--TTTRC ARQ-
C8C423_LAGAC     MARNR-CRSP --SQSRCR-R ---P-RRRCL --RRRRCCRR --RR--RVCC RRY--TTTRC AR--
F5CBC9_CEPCM     MARNR-CRSP --SQSRCR-R ---P-RRRCR --RRRRCCRR --RR--RVCC RRY--ATTRC ARQ-
F5CBC8_LAGAL     MARNR-CRSP --SQSRCR-R ---P-RRRCR --RRRRCCRR --RR--RVCC RRY--TTTRC ASQ-
C8C421_TURTR     MARNR-CRSP --SQSRCR-R ---P-RRRCR --RRRLCCRR --RR--RVCC RRY--TTTRC ARQ-
F5CBC0_TURAD     MARNR-CRSP --SQSRCR-R ---P-RRRCR --RRRLCCRR --RR--RVCC RRY--TTTRC ARQ-
B4YVN1_STECO     MARNR-CRSP --SQSRCR-R ---P-RRRCR --RRRLCCRR --RR--RVCC RRY--TTTRC ARQ-
F5CBC1_STELO     MARNR-CRSP --SQSRCR-R ---P-RRRCR --RRRRCCRR --RR--RVCC RRY--TTTRC AR--
F5CBC5_LAGHO     MARNR-CRSP --SQSRCR-R ---P-RRRCR --RRRRCCRR --RR--RVCC RRY--TTTRC AR--
F5CBB9_DELCA     MARNR-CRSP --SQSRCR-R ---P-RRRCR --RRRRCCRR --RR--RVCC RRY--TTTRC AR--
F5CBC3_STEAT     MARNR-CRSP --SQSRCR-R ---P-RRRCR --RRRRCCRR --RR--RVCC RRY--TTTRC AR--
F5CBC4_SOUCH     MARNR-CRSP --SQSRCR-R ---P-RRRCR --RRRRCCRR --RR--RVCC RRY--TTTRC ARQ-
F5CBD1_9CETA     MARNR-CRSP --SQSRCR-R ---P-RRRCR --RRRRCCRR --RR--RVCC RRY--TTTRC ARQ-
F5CBC7_LAGOL     MARNR-CRSP --SQSRCR-R ---P-RRRCR --RRRRCCRR --RR--RVCC RRY--TTTRC AR--
B4YVM9_STEBR     MARNR-CRSP --SQSRCR-R ---P-RRRCR --RRRRCCRR --RR--RVCC RRY--TTTRC ARQ-
F5CBC2_STEFR     MARNR-CRSP --SQSRCR-R ---P-RRRCR --RRRRCCRR --RR--RVCC RRY--TTTRC AR--
F5CBC6_SOTFL     MARNR-CRSP --SQSRCR-R ---P-RRRCR --RRRRCCRR --RR--RVCC RRY--TTTRC AR--
C8C422_LISBO     MARNR-CRSP --SQSRCR-R ---P-RRRCR --RRRRCCRR --RR--RVCC RRY--TTTRC ARQ-
B4YVN0_DELDE     MARNR-CRSP --SQSRCR-R ---P-RRRCR --RRRRCCRR --RR--RVCC RRY--TTTRC AR--
C8C428_NEOPH     MARNR-CRSP --SQSRGR-C ---P-RRRYR -SKRRRCCQR --RR--RVCR RRY---TRRC ARQ-
C8C427_9CETA     MARNR-CRSP --SQSRGR-C ---P-RRRYR -SKRRRCCQR --RR--RVCR RRY---TRRC ARQ-
B4YVM6_PHOPH     MARNR-CRSP --SQSRGR-C ---P-RRRYR -SKRRRCCQR --RR--RVCR RRY---TRRC ARQ-
F5CIP3_LIPVE     MARNR-CRSP --SQSRGR-R ---P-RRKYR -SRRRRCCQR --RR--RVCC RRY--TTMRC AKQ-
C8C430_DELLE     MARNR-CRSP --SQSRGR-R ---P-RRRYR -SKRRRCCQR --RR--RVCC RRY--TTTRC ARQ-
C8C429_MONMO     MARTR-CRSP --SQSRGR-R ---P-RRRYR -SKRRRCCQR --RR--RVCC RRY--TTTRC ARQ-
A8IYB2_PONBL     MARNR-CRSP --SQNRGR-R ---P-RRRYR -SRRRRCCQR --RR--RVCC RRY--TSVRC ARQ-
A8IYA9_INIGE     MARNR-CRSP --SQSRGR-R ---P-RRRYR -SRRRRCCQR --RR--RVCC RRY--TTVRC ARQ-
F7VJK6_LOXAF     MARYRCCRSR --SRSRCRSR ---R-RRRSH -RRRRRCARR --RRRTRRGC RRR--YSLRR RRY-
HSP1_PONPY       MARYRCCRSQ --SQSRCC-R ---R-RQRCH -RRRRRCCQT --RRRAMRCC RRR--YRLRC RRH-
H2NQ55_PONAB     MARYRCCRSQ --SQSRCC-R ---R-RQRCH -RRRRRCCQT --RRRAMRCC RRR--YRLRC RRH-
HSP1_GALVR       MARYRCCR-- --SRSRCR-R ---R-RRSCR --RRRRCRRR --RA--RRSC RRR--YSLRC CRRY
HSP1_SAGIM       MARYRCCRSQ --SRSRCY-R ---Q-RRRGR -RRRRRTCRR --RRA-SRCC RRR--YKLTC RRY-
A0A2R8PK40_CALJA MARYRCCRSQ --SRSRCY-R ---Q-RRRGR -RRRRRSCRR --RRA-SRCC RRR--YKLPC RRY-
HSP1_ALOSE       MARYRCCRSR SLSRSRCY-R ---Q-RPRCR -RRRRRSCRR -PRA--SRCC RRR--YRLRR RRY-
HSP1_SAISC       MARYRCCRSR --SRSRCY-R ---R-RRRCR -TRRRRCCRR --RRA-RRCC RRR--YKLRC RRY-
A0A2K6TB39_SAIBB MARYRCCRSR --SRSRCY-R ---R-RRRCR -TRRRRCCRR --RRA-RRCC RRR--YKLRC RRY-
A0A2K5R6E5_CEBCA MARYRCCRSR --SRSRCY-R ---Q-RRRCR -RRRRRCRSR --RA--RRCC RRR--YRLRC RRY-
HSP1_RABIT       MVRYRCCRSQ --SRSRCR-R ---R-RRRCR -RRRRRCCQR --RRV-RKCC RRT--YTLRC RRY-
HSP1_CAVPO       MARYRCCRSP --SRSRCR-R ---R-RRRFY -RRRRRCHRR --RR---RCC RRR--YTRRC KRY-
HSP1_COLGU       MARYRCRRSQ --SRSRCC-R ---Q-RRRCR -RRRRQRFRA --RKRAMRCC HRR--YRLRC RRY-
F7VJK5_ERIEU     MARYRCCRSQ --SRSRCS-R RRYR-RRRCR -RRRRRSCRR --RRRR-ACC RYR--Y---- RRY-
C3U1R1_9MURI     MARYRCCRSK --SRSRCR-R ---R-RRRCR -RRRRRCCQR --RR--RRCC RRRRSYTIRC KKY-
HSP1_MOUSE       MARYRCCRSK --SRSRCR-R ---R-RRRCR -RRRRRCCRR --RR--RRCC RRRRSYTIRC KKY-
C3U1R5_MUSSP     MARYRCCRSK --SRSRCR-R ---R-RRRCR -RRRRRCCRR --RR--RRCC RRRRSYTIRC KKY-
C3U1Q6_MUSMB     MARYRCCRSK --SRSRCR-R ---R-RRRCR -RRRRRCCRR --RR--RRCC RRRRSYTIRC KKY-
C3U1Q9_MOUSE     MARYRCCRSK --SRSRCR-R ---R-RRRCR -RRRRRCCRR --RR--RRCC RRRRSYTIRC KKY-
C3U1R0_MOUSE     MARYRCCRSK --SRSRCR-R ---R-RRRCR -RRRRRCCRR --RR--RRCC RRRRSYTIRC KKY-
C3U1Q8_MUSMC     MARYRCCRSK --SRSRCR-R ---R-RRRCR -RRRRRCCRR --RR--RRCC RRRRSYTIRC KKY-
C3U1R4_MUSSI     MARYRCCRSK --SRSRCR-R ---R-RRRCR -RRRRRCCRR --RR--RRCC RRRRSYTIRC KKY-
C3U1Q7_MUSCO     MARYRCCRSK --SRSRCR-R ---R-RRRCR -RRRRRCCRR --RR--RRCC RRRRSYTIRC KKY-
C3U1R3_MUSMA     MARYRCCRSK --SRSRCR-R ---R-RRRCR -RRRRRCCRR --RR--RRCC RRRRSYTIRC KKY-
C3U1R2_MUSPA     MARYRCCRSK --SRSRCR-R ---R-RRRCR -RRRRRCCRR --RR--QRCC RRRRSYTIRC KKY-
HSP1_RAT         MARYRCCRSK --SRSRCR-R ---R-RRRCR -RRRRRCCRR --RR--RRCC RRRRSYTFRC KRY-
C3U1S7_APOSY     MARYRCCRSK --SRSRCR-R ---R-RRRCR SRRRRRCCRR --RR--RRCC RRRRSYTIRC KRY-
HSP1_NASLA       MAKSRCCGSQ --SRSRCC-R ---P-RQRCR -RRRRRSCRA --RRRAMRCC RRR--YRLRC RRY-
HSP1_TRAJO       MARYRRCRSQ --SRSRCC-R ---P-RRRCR -RRRRQSCRA --RRRATRCC RRR--YRLRC RRY-
HSP1_TRAPH       MARYRCCRSQ --SRSRCC-R ---P-RRRCR -RRRRRSCRA --RRRATRCC RRR--YRL-S RRY-
L0CM34_TRAPH     MARYRCCRSQ --SRSRCC-R ---P-RRRCR -RRRRRSCRA --RRRATRCC RRR--YRL-S RRY-
HSP1_TRACR       MARYRCCRSQ --SRSRCC-R ---P-RRRCR -RRRRRSCRA --RRRATRCC RRR--YRLRS RRY-
HSP1_TRAOB       MARYRCCRSQ --SRSRCC-R ---P-RRRCR -RRRRRSCRA --RRRATRCC RRR--YRLRS RRY-
L0CM39_9PRIM     MARYRCCRSQ --SRSRCC-R ---P-RRRCR -RRRRRSCRA --RRRATRCC RRR--YRLRS RRY-
L0CNC1_TRAPH     MARYRCCRSQ --SRSRCC-R ---P-RRRCR -RRRRRSCRA --RRRATRCC RRR--YRLRS RRY-
HSP1_SEMEN       MARYRRCRSQ --SRSRCC-R ---P-RRRCR --RRRRSCRA --RRRATRCC RRR--YRLRC RRY-
HSP1_TRAVT       MARYRRCRSQ --SRSRCC-R ---P-RRRCR --RRRRSCRA --RRRATRCC RRR--YRLRC RRY-
HSP1_TRAPL       MARYRCCRSQ --SRSRCC-R ---P-RRRCR -RRRRRSCRA --RRRATRCC RRR--YRLRC RRY-
HSP1_TRAGE       MARYRCCRSQ --SRSRCC-R ---P-RRRCR -RRRRRSCRA --RRRATRCC RRR--YRLRC RRY-
HSP1_TRAFR       MARYRCCRSQ --SRSRCC-R ---P-RRRCR -RRRRRSCRA --RRRATRCC RRR--YRLRC RRY-
S4SNK2_9PRIM     -------RSQ --SRSRCC-R ---P-RRRCR --RRRRSCRA --RRRATRCC RRR--YRLRC RRY-
S4SLN4_9PRIM     -------RSQ --SRSRCC-R ---P-RRRCR --RRRRSCRA --RRRATRCC RRR--YRLRC RRY-
F7VJK8_OTOGA     MARYRCCRSQ --SRSRCR-R ---R-RRRCR -RRRRRCCRR --RR--RRCC RRR--YRLRC RRY-
HSP1_PILBA       MARYRCCRSQ --SRSRCC-R ---R-RRRCR -RRRRQRCRA --RRTAMRCC RRR--YRRRC RRY-
HSP1_MACMU       MARYRCCRSQ --SRSRCC-R ---R-RRRCR -RRRRRRCRA --RRRAMRCC RRR--YRLRC RRY-
HSP1_PAPCY       MARYRCCRSQ --SRSRCC-R ---R-RRRCR -RRRRRRCRA --RRRAMRCC RRR--YRLRC RRY-
A0A096MLL5_PAPAN MARYRCCRSQ --SRSRCC-R ---Q-RRRCR -RRRRRRCRA --RRRAMRCC RRR--YRLRC RRY-
HSP1_HYLLA       MARYRCCRSQ --SRSRCY-R ---R-GQRSR -RRRRRSCQT --RRRAMRCC RPR--YRLRR RRH-
HSP1_HUMAN       MARYRCCRSQ --SRSRYY-R ---Q-RQRSR -RRRRRSCQT --RRRAMRCC RPR--YRPRC RRH-
HSP1_GORGO       MARYRCCRSQ --SRSRCY-R ---Q-RQTSR -RRRRRSCQT --QRRAMRCC RRR--NRLRR RKH-
HSP1_PANTR       MARYRCCRSQ --SRSRCY-R ---Q-RQRSR -RRKRQSCQT --QRRAMRCC RRR--SRMRR RRH-
HSP1_PANPA       MARYRCCRSQ --SRSRCY-R -----QRRSR -RRKRQSCQT --QRRAMRCC RRR--SRLRR RRH-
```

Supplemental Figure 2. Alignment of Eutherian P1 type sperm protamines.

```
                       10         20         30         40         50         60
PRM2_RATTU  MVRYRMRSPS ESPHQGPGQD HESEEQG--- -QGQELNPER VEDYGRTHRG --HHRHRRCS
PRM2_MOUSE  MVRYRMRSPS EGPHQGPGQD HEREEQG--- -QGQGLSPER VEDYGRTHRG HHHHRHRRCS
PRM2_RATFU  MVRYRMRSPS EGPHQGPGQD HEREEQG--- -QGQELSPER VEDYGRTHRG --HHRHRRCS
PRM2_ALOSE  MVRYHVRSPS ERPHREYRQL VNGQEQGRHG QEEQGMSAEG VEGYGRTHQG CYGYRRRLCS
PRM2_CALJA  MVRYRVRSPS ERPHEEYRQL VNWQEQGRNG QEEQGLSAEG GEVYGRTHQG YSSYRRRRCS
PRM2_SEMEN  MVRYRMRSLS ERPHEVHGQQ VYGQEQGHNG QEEQGLSPEH VEVYERTHQG YSHHRRRRCS
PRM2_ERYPA  MVRYRTRSLS ERPHEVHGQQ VHGQDQGHNG QEEQGLSPEH VEVYERTHQG HSHHRRRRCS
PRM2_MACNE  MVRYRMRSLS ERPHEVHGQQ VHGQDQGHNG QEEQGLNPEH VEVYERTHRG HSHHRRRRCS
PRM2_MACFU  MVRYRMRSLS ERPHEVHGQQ VHGQDQGHNG QEEQGLNPEH VEVYERTHRG HSHHRRRRCS
PRM2_MACMU  MVRYRMRSLS ERSHEVHGQQ VHGQDQGHNG QEEQGLNPEH VEVYERTH-G HSHYRRRHCS
PRM2_GORGO  MVRCRVRSPS ERSHEVYRQQ LHGQEQGHHG QEEQGLSPEH VEVYERTH-G HSHYRRRHCS
PRM2_PANPA  MVRYRVRSPS EPSHEVYRQQ LHGQEQGHHG QEEQGLSPEH VEVYERTH-G HSHYRRRHCS
PRM2_PANTR  MVRYRVRSPS EPSHEVYRQQ LHGQEQGHHG QEEQGLSPEH VEVYERTH-G HSHYRRRHCS
PRM2_HUMAN  MVRYRVRSLS ERSHEVYRQQ LHGQEQGHHG QEEQGLSPEH VEVYERTH-G QSHYRRRHCS
PRM2_PONPY  MVRYCVRSLS ERSHEVYGQQ LHGQEQGHHD QEEQGLSPEQ VEVYERTQ-G HSHYRRRHCS
PRM2_HYLLA  MVRYCVRSLS ERSHEVYGQQ LRGQEQGHHG QEEQGLSPED VEVYERTH-G HSHYRRRHCS
                       70         80         90        100        110        120
PRM2_RATTU  RKRLHRIHKR R-RSCRRRRR HSCCHRRRHR RGCRRSRRRR RCRCRKCRRQ CH
PRM2_MOUSE  RKRLHRIHKR R-RSCRRRRR HSCRHRRRHR RGCRRSRRRR RCRCRKCRRH HH
PRM2_RATFU  RKRLHRIHKR R-RSCRRRRR HSCCHRRRHR RGCRRSRRRR RCKCRKCRRH CH
PRM2_ALOSE  RRRLYRVHRR QRRSCRRRC- --CRYRRRNR RGCRT-RRRT ------CRRH --
PRM2_CALJA  RRRRYRIHRR RSRSCRRRRR RSCRYRRRPR RGCRSRRRRR ------CRRY --
PRM2_SEMEN  RRRLYRIHRR RHRSCRRRRR RSCRHRRRHR RGCRT-RRRR ------CRRY --
PRM2_ERYPA  QRRLHRIHRR RHRSCRRRRR RSCRHRRRHR RGCRT-RRRR ------CRRY --
PRM2_MACNE  RRRLHRIHRR RHRSCRRRRR RSCRHRRRHR RGCRT-RRRR ------CRRH --
PRM2_MACFU  RRRLHRIHRR RHRSCRRRRR RSCRHRRRHR RGCRT-RRRR ------CRRH --
PRM2_MACMU  RRRLHRIHRR RHRSCRRRRR RSCRHRRRHR RGCRT-RRRR ------CRRH --
PRM2_GORGO  RRRLRRIHRQ QHRSCRRRKR RSCRHRRRHR KGCRT-RRRT ------CRRH --
PRM2_PANPA  RRRLRRIHRQ QHRSCRRRKR RSCRHRRRHR RGCRT-RRRT ------CRKH --
PRM2_PANTR  RRRLRRIHRQ QHRSCRRRKR RSCRHRRKHR RGCRT-RRRT ------CRRH --
PRM2_HUMAN  RRRLHRIHRR QHRSCRRRKR RSCRHRRRHR RGCRT-RKRT ------CRRH --
PRM2_PONPY  RRRLHRIHRQ QHRSCKRRRR HSCRHRRKHR RGCRT-RRRT ------CRRH --
PRM2_HYLLA  RRRLHRIHRQ QHRSCGRRRR RSCRQRRRHR RGCRT-RRRR ------CRRH --
```

Supplemental Figure 3. Alignment of Eutherian P2 type sperm protamines.

```
                           10         20         30         40         50         60
HSP1_PERGU        MASYR-NSRS RSRSRF-RRR RRGRSRVRGR DARQGRSSR- RRRRGKGR-- --AHSGKKGR
HSP1_ISOMA        MASYR-NSRS RSRSRF-RRR RGRRSRVRGR DARQGRSSR- RRRRGKGR-- --AHSGKKGR
HSP1_MONDO        MARYRRRSRS RSRSRYGRRR RRSRS----R RRRSRRRRR- ---RRGRR-- ---GRGYHRR
HSP1_DIDVI        MARYRRRSRS RSRSRYGRRR RRSRS----R RRRSRRRRR- ---RRGRR-- ---GRGYHRR
HSP1_DROGL        MVRYRRHSRS RSRSRY-RRR RRRR--LRNR RRRYRRSRRG RRRRRRGS-- ---RRGYSRR
HSP1_NOTTY        MARYR-HSRS RSRSRY-RRR RRRRSRYRSQ RRRYRRHRRS GRRRRRGR-- ---RRGY-RR
HSP1_PHACI        MARYR-HSRS RSRSRY-QRR RRRRSRYRSQ RRRYRRRRGS RRRRRRGR-- ---RRGY-RR
HSP1_HYPMS        MARYR-HSRS RSRSRY-RRR RRRRSRYRGR RRRYRRSRR- RRRSRRGR-- ---RGYYRRR
HSP1_LAGHI        MARYR-HSRS RSRSGY-RRQ RRRRSRYRSR RRRYRRR-Q- -RRSRRGR-- ---RRGYSRR
HSP1_DENDO        MARYR-HSRS RXRSRY-RRR RRXRSRYRSX RRRYRGRRR- RRSRRGRR-- ---RRGYSRX
HSP1_AEPRU        MARYR-HSRS RSRSRY-RRR RRRRSRYRSR RRRYRGSRR- RRRSRRRR-- ---RRGYSRR
HSP1_PSECU        MARYR-HSRS RSRSRYRRRR RRRRSRYRGR RRRYRRSRR- RRRRGRRRGN CLGRRGYRRR
HSP1_TRIVU        MARYR-HSRS RSRSRYRRRR RRRRSRYRSR RRRYRRSRR- -RRRRGRR-- ---RRGYSRR
HSP1_CAEFU        MARYR-HSRS RSRSRYRRRR RRRRSRYRSR RRRYRRSRR- --RRRRGR-- ---RRGYSRR
HSP1_POTLO        MARYR-HSRS RSRXRY-RRR RRRRSRYRSR RRRYRGSRR- SRSRRRGR-- ---RRGYSRR
HSP1_LAGFA        MARYR-HSRS RSRSRY-RRR RRRRSRYRSR RRRYRGSRR- SRSRRRGR-- ---RRGYSRR
HSP1_BETPE        MARYR-HSRS RSRSRY-RRR RRRRSRYRSR RRRYRGSRR- RRSRRRGR-- ---RRGYSRR
HSP1_ONCFR        MARYR-HSRS RSRSXY-RRR XRRRSRYRSR RRRYRGRRR- -RRSRRGR-- ---RRGYSRR
HSP1_MACRU        MARYR-HSRS RSRSRY-RRR RRRRSRYRSQ RRRYRGRRR- -RRSRRGR-- ---RRGYSRR
HSP1_PETCN        MARYR-HSRS RSRSRY-RRR RRRRSRYRSR RRRXRR--R- RRSRRGRR-- ---RRGYSRR
HSP1_MACPA        MARYR-HSRS RSRSRY-RRR RRRRSRYRSR RRRYRGRRR- -XRSRRGR-- ---RRGYSRR
HSP1_MACEU        MARYR-HSRS RSRSRY-RRR RRRRSRYRSR RRRSRGRRR- RRSRRGRR-- ---RRGYSRR
HSP1_MACAG        MARYR-HSRS RSRSRY-RRR RRRRSRYRSR RRRSRGRRR- -RRSRRGR-- ---RRGYSRR
HSP1_DORVA        MARYR-HSRS RSRSRY-RRR RRRRSRYRSR RRXYRGRRR- RRSRRGRR-- ---RRGYSRR
HSP1_PETXA        MARYR-HSXS RSRSRY-RRR RRRRSRYRSR RRRYRGRRR- RRSRRGRR-- ---RRGYSRR
HSP1_THYST        MARYR-HSRS RSRSRY-RRR RRRRSRYRSR RRRYRGRRR- RRSRRGRR-- ---RRGYSCR
HSP1_MACGI        MARYR-HSRS RSRSRY-RRR RRRRSRYRSR RRRYRGRRR- RRSRRGRR-- ---RRGYSRR
HSP1_DORMU        MARYR-HSRS RSRSRY-RRR RRRRSRYRSR RRRYRGRRR- RRSRRGRR-- ---RRGYSRR
HSP1_WALBI        MARYR-HSRS RSRSRY-RRR RRRRSRYRSR RRRYRGRRR- RRSRRGRR-- ---RRGYSRR
HSP1_MACRG        MARYR-HSRS RSRSRY-RRR RRRRSRYRSR RRRYRGRRR- RRSRRGRR-- ---RRGYSRR
HSP1_ONYUN        MARYR-HSRS RSRSRY-RRR RRRRSRYRSR RRRYRGRRR- -RRSRRGR-- ---RRGYSRR
HSP1_DENGO        MARYR-HSRS RSRSRY-RRR RRRRSRYRSR RRRYRGRRR- -RRSRRGR-- ---RRGYSRR
HSP1_SETBR        MARYR-HSRS RSRSRY-RRR RRRRSRYRSR RRRYRGRRR- -RRSRRGR-- ---RRGYSRR
HSP1_DASHA        MARYRRRSRS RSRSRY-RRR RRRRSRGR-R RRTYRRSRR- HSRRRRGR-- ---RRGYSRR
HSP1_SARHA        MARYRRRSRS RSRSRY-RRR RRRRSRGR-R RRTYRRSRR- HSRRRRGR-- ---RRGYSRR
HSP1_DASMA        MARYRRRSRS RSRSRY-RRR RRRRSRGR-R RRTYRRSRR- HSRRRRGR-- ---RRGYSRR
HSP1_DASSP        MARYRRHSRS RSRSRY-RRR RRRRSRGR-R RRTYRRSRR- HSRRRRGR-- ---RRGYSRR
HSP1_NEOLO        MARYRRHSRS RSRSRY-RRR RRRRSRGR-R RRTYRRSRR- HSRRRRGR-- ---RRGYSRR
HSP1_DASVI        MARYRRHSRS RSRSRY-RRR RRRRSRGR-R RRTYRRSRR- HSRRRRGR-- ---RRGYSRR
HSP1_PHADO        MARYRRHSRS RSRSRY-RRR RRRRSRGR-R RRTYRRSRR- HSRRRRGR-- ---RRGYSRR
HSP1_DASAL        MARYRRHSRS RSRSRY-RRR RRRRSRGR-R RRTYRRSRR- HSRRRRGR-- ---RRGYSRR
HSP1_DASGE        MARYRRHSRS RSRSRY-RRR RRRRSRGR-R RRTYRRSRR- HSRRRRGR-- ---RRGYSRR
HSP1_DASRO        MARYRRHSRS RSRSRY-RRR RRRRSRHRNR RRTYRRSRR- HSRRRRGR-- ---RRGYSRR
HSP1_PSEBA        MARYRRHSRS RSRSRY-RRR RRRRSRHRNR RRTYRRSRR- HSRRRRGR-- ---RRGYSRR
HSP1_PARAP        MARYRRHSRS RSRSRY-RRR RRRRSRHRNR RRTYRRSRR- HSRRRRGR-- ---RRGYSRR
HSP1_PSENI        MARYRRHSRS RSRSRY-RRR RRRRSRHRNR RRTYRRSRR- HSRRRRGR-- ---RRGYSRR
HSP1_DASBY        MARYRRHSRS RSRSRY-RRR RRRRSRHRNR RRTYRRSRR- HSRRRRGR-- ---RRGYSRR
HSP1_MYOME        MARYRRHSRS RSRSRY-RRR RRRRSRHRNR RRTYRRSRR- HSRRRRGR-- ---RRGYSRR
HSP1_MYOWA        MARYRRHSRS RSRSRY-RRR RRRRSRHRNR RRTYRRSRR- HSRRRRGR-- ---RRGYSRR
HSP1_PSEMD        MARYRRHSRS RSRSRY-RRR RRRRSRHRNR RRTYRRSRR- HSRRRRGR-- ---RRGYSRR
HSP1_PSEWO        MARYRRHSRS RSRSRY-RRR RRRRSRHRNR RRTYRRSRR- HSRRRRGR-- ---RRGYSRR
HSP1_SMIGR        MARYRRHSRS RSRSRY-RRR RRRRSRHHNR RRTYRRSRR- HSRRRRRR-- ---RRGYSRR
HSP1_SMIBI        MARYRRHSRS RSRSRY-RRR RRRRSRHHNR RRTYRRSRR- HSIRRRGR-- ---RRGYSRR
HSP1_SMILO        MARYRRHSRS RSRSRY-RRR RRRRSRHHNR RRTYRRSRR- HSRRRRGR-- ---RRGYSRR
HSP1_ANTFL        MARYRRHSRS RSRSRY-RRR RRRRSRHHNR RRTYRRSRR- HSRRRRGR-- ---RRGYSRR
HSP1_NINTI        MARYRRHSRS RSRSRY-RRR RRRRSRHHNR RRTYRRSRR- HSRRRRGR-- ---RRGYSRR
HSP1_NINYV        MARYRRHSRS RSRSRY-RRR RRRRSRHHNR RRTYRRSRR- HSRRRRGR-- ---RRGYSRR
HSP1_PARRT        MARYRRHSRS RSRSRY-RRR RRRRSRHHNR RRTYRRSRR- HSRRRRGR-- ---RRGYSRR
HSP1_SMILE        MARYRRHSRS RSRSRY-RRR RRRRSRHHNR RRTYRRSRR- HSRRRRGR-- ---RRGYSRR
HSP1_PHATA        MARYRRHSRS RSRSRY-RRR RRRRSRHHNR RRTYRRSRR- HSRRRRGR-- ---RRGYSRR
HSP1_SMIPS        MARYRRHSRS RSRSRY-RRR RRRRSRHHNR RRTYRRSRR- HSRRRRGR-- ---RRGYSRR
HSP1_ANTBE        MARYRRHSRS RSRSRY-RRR RRRRSRHHNR RRTYRRSRR- HSRRRRGR-- ---RRGYSRR
HSP1_MICHA        MARYRRHSRS RSRSRY-RRR RRRRSRHHNR RRTYRRSRR- HSRRRRGR-- ---RRGYSRR
HSP1_ANTGO        MARYRRHSRS RSRSRY-RRR RRRRSRHHNR RRTYRRSRR- HSRRRRGR-- ---RRGYSRR
HSP1_ANTMI        MARYRRHSRS RSRSRY-RRR RRRRSRHHNR RRTYRRSRR- HSRRRRGR-- ---RRGYSRR
HSP1_ANTNA        MARYRRHSRS RSRSRY-RRR RRRRSRHHNR RRTYRRSRR- HSRRRRGR-- ---RRGYSRR
HSP1_ANTSW        MARYRRHSRS RSRSRY-RRR RRRRSRHHNR RRTYRRSRR- HSRRRRGR-- ---RRGYSRR
HSP1_MURME        MARYRRHSRS RSRSRY-RRR RRRRSRHHNR RRTYRRSRR- HSRRRRGR-- ---RRGYSRR
HSP1_SMIDO        MARYRRHSRS RSRSRY-RRR RRRRSRHHNR RRTYRRSRR- HSRRRRGR-- ---RRGYSRR
HSP1_SMIHI        MARYRRHSRS RSRSRY-RRR RRRRSRHHNR RRTYRRSRR- HSRRRRGR-- ---RRGYSRR
HSP1_SMIMA        MARYRRHSRS RSRSRY-RRR RRRRSRHHNR RRTYRRSRR- HSRRRRGR-- ---RRGYSRR
HSP1_SMIOO        MARYRRHSRS RSRSRY-RRR RRRRSRHHNR RRTYRRSRR- HSRRRRGR-- ---RRGYSRR
HSP1_SMIYO        MARYRRHSRS RSRSRY-RRR RRRRSRHHNR RRTYRRSRR- HSRRRRGR-- ---RRGYSRR
HSP1_PLAMM        MARYRRHSRS RSRSRY-RRR RRRRSRHHNR RRTYRRSRR- HSRRRRGR-- ---RRGYSRR
HSP1_SMIAR        MARYRRHSRS RSRSRY-RRR RRRRSRHHNR RRTYRRSRR- HSRRRRGR-- ---RRGYSRR
HSP1_DASCR        MARYRRHSRS RSRSRY-RRR RRRRSRHHNR RRTYRRSRR- HSRRRRGR-- ---RRGYSRR
HSP1_ANTLE        MARYRRHSRS RSRSRY-RRR RRRRSRHHNR RRTYRRSRR- HSRRRRGR-- ---RRGYSRR
HSP1_ANTST        MARYRRHSRS RSRSRY-RRR RRRRSRHHNR RRTYRRSRR- HSRRRRGR-- ---RRGYSRR
HSP1_MURLO        MARYRRHSRS RSRSRY-RRR RRRRSRHHNR RRTYRRSRR- HSRRRRGR-- ---RRGYSRR
HSP1_MYRFA        MARYRRHSRS RSRSRY-RRR RRRRSRHHNR RRTYRRSRR- HSRRRRGR-- ---RRGYSRR
HSP1_NINRI        MARYRRHSRS RSRSRY-RRR RRRRSRHHNR RRTYRRSRR- HSRRRRGR-- ---RRGYSRR
HSP1_SMIGA        MARYRRHSRS RSRSRY-RRR RRRRSRHHNR RRTYRRSRR- HSRRRRGR-- ---RRGYSRR
HSP1_SMIGI        MARYRRHSRS RSRSRY-RRR RRRRSRHHNR RRTYRRSRR- HSRRRRGR-- ---RRGYSRR
HSP1_PHACL        MARYRRHSRS RSRSRY-RRR RRRRSRHHNR RRTYRRSRR- HSRRRRGR-- ---RRGYSRR
HSP1_SMIMU        MARYRRHSRS RSRSRY-RRR RRRRSRHHNR RRTYRRSRR- HSRRRRGR-- ---RRGYSRR
HSP1_SMIVI        MARYRRHSRS RSRSRY-RRR RRRRSRHHNR RRTYRRSRR- HSRRRRGR-- ---RRGYSRR
HSP1_THYCY        MARYRRHSRS RSRSRY-RRR RRRRSRHHNR RRTYRRSRR- HSRRRRGR-- ---RRGYSRR
HSP1_SMIAI        MARYRRHSRS RSRSRY-RRR RRRRSRHHNR RRTYRRSRR- HSRRRRGR-- ---RRGYSRR
HSP1_SMICR        MARYRRHSRS RSRSRY-RRR RRRRSRHHNR RRTYRRSRR- HSRRRRGR-- ---RRGYSRR
HSP1_SMIDL        MARYRRHSRS RSRSRY-RRR RRRRSRHHNR RRTYRRSRR- HSRRRRGR-- ---RRGYSRR
HSP1_ANTLA        MARYRRHSRS RSRSRY-RRR RRRRSRHHN- RRTYRRSRR- HSRRRRGR-- ---RRGYSRR
HSP1_PLAMS        MARCRRHSRS RSRSRN-QCQ RRRRRRY-NR RRTYRRSRR- HSRRRRGR-- ---RRGCSRR
HSP1_PLAIN        MARSRRHSRS RSRSR--NQC QRRRRRTYNR RRTMREKPR- HSRRRRVR-- ---RRGCSCR
HSP1_PLAGI        MARCRRHSRS RSRSRN-QCQ RRRR-RHYNR RRTYRRSRR- HSRRRRVR-- ---RRGCSCR
HSP1_PLATE        MARCRRHIRS RSRSRN-QCQ RRRRRSHYNR RRTYRRSRR- HSRRRRVR-- ---RRGCSCR
                           70         80         90
HSP1_PERGU        RSG-----SR RRKRNNNTEN K
HSP1_ISOMA        RSG-----SR RRKRNNENK- -
HSP1_MONDO        SPHRR---RR RRRR------ -
HSP1_DIDVI        SPHRR---RR RRRR------ -
HSP1_DROGL        RYQSR---RR RRRRY----- -
HSP1_NOTTY        RYH------S HRRRY----- -
HSP1_PHACI        RYS-----RR --RRY----- -
HSP1_HYPMS        RYS-----RR RRRRYYY--- -
HSP1_LAGHI        RYSRRRYSRR RRRRY----- -
HSP1_DENDO        RYS-----RR RRRRY----- -
HSP1_AEPRU        RYS------R RRRRY----- -
HSP1_PSECU        RYS-----RR RRRRYY---- -
HSP1_TRIVU        RYS-----RR GRRRY----- -
HSP1_CAEFU        RYS-----RR RRRRY----- -
HSP1_POTLO        RYS------R RRRRY----- -
HSP1_LAGFA        RYS-----RR RRRRY----- -
HSP1_BETPE        RYS-----RR RRRRY----- -
HSP1_ONCFR        RYS-----RR RRRRY----- -
HSP1_MACRU        RYS------R RRRRY----- -
HSP1_PETCN        RYS-----RR RRRRY----- -
HSP1_MACPA        RYS-----RR RRRRY----- -
HSP1_MACEU        RYS-----RR RRRRY----- -
HSP1_MACAG        RYS-----RR RRRRY----- -
HSP1_DORVA        RYS-----RR RRRRY----- -
HSP1_PETXA        RYS-----RR RRRRY----- -
HSP1_THYST        RYS-----RR RRRRY----- -
HSP1_MACGI        RYS------R RRRRY----- -
HSP1_DORMU        RYS-----RR RRRRY----- -
HSP1_WALBI        RYS-----RR RRRRY----- -
HSP1_MACRG        RYS-----RR RRRRY----- -
HSP1_ONYUN        RYS-----RR RRRRY----- -
HSP1_DENGO        RYS-----RR RRRRY----- -
HSP1_SETBR        RYS-----RR RRRRY----- -
HSP1_DASHA        RYS-----RR GRRR------ -
HSP1_SARHA        RYS-----RR GRRRY----- -
HSP1_DASMA        RYS-----RR GRRRY----- -
HSP1_DASSP        RYS-----RR GRRRY----- -
HSP1_NEOLO        RYS-----RR GRRRY----- -
HSP1_DASVI        RYS-----RR GRRRY----- -
HSP1_PHADO        RYS-----RR GRRRY----- -
HSP1_DASAL        RYS-----RR GRRRY----- -
HSP1_DASGE        RYS-----RR GRRRY----- -
HSP1_DASRO        RYS-----RR GRRRY----- -
HSP1_PSEBA        RYS-----RR GRRR------ -
HSP1_PARAP        RYS-----RR GRRRY----- -
HSP1_PSENI        RYS-----RR GRRRY----- -
HSP1_DASBY        RYS-----RR GRRRY----- -
HSP1_MYOME        RYS-----RR GRRRY----- -
HSP1_MYOWA        RYS-----RR GRRRY----- -
HSP1_PSEMD        RYS-----RR GRRRY----- -
HSP1_PSEWO        RYS-----RR GRRRY----- -
HSP1_SMIGR        RYS-----RR GRRRY----- -
HSP1_SMIBI        RYS-----RR GRRRY----- -
HSP1_SMILO        RYS-----RR -RRRY----- -
HSP1_ANTFL        RYS-----RR GRRRY----- -
HSP1_NINTI        RYS-----RR GRRRY----- -
HSP1_NINYV        RYS-----RR GRRRY----- -
HSP1_PARRT        RYS-----RR GRRRY----- -
HSP1_SMILE        RYS-----RR GRRRY----- -
HSP1_PHATA        RYS-----RR GRRRY----- -
HSP1_SMIPS        RYS-----RR GRRRY----- -
HSP1_ANTBE        RYS-----RR GRRRY----- -
HSP1_MICHA        RYS-----RR GRRRY----- -
HSP1_ANTGO        RYS-----RR GRRRY----- -
HSP1_ANTMI        RYS-----RR GRRRY----- -
HSP1_ANTNA        RYS-----RR GRRRY----- -
HSP1_ANTSW        RYS-----RR GRRRY----- -
HSP1_MURME        RYS-----RR GRRRY----- -
HSP1_SMIDO        RYS-----RR GRRRY----- -
HSP1_SMIHI        RYS-----RR GRRRY----- -
HSP1_SMIMA        RYS-----RR GRRRY----- -
HSP1_SMIOO        RYS-----RR GRRRY----- -
HSP1_SMIYO        RYS-----RR GRRRY----- -
HSP1_PLAMM        RYS-----RR GRRRY----- -
HSP1_SMIAR        RYS-----RR GRRRY----- -
HSP1_DASCR        RYS-----RR GRRRY----- -
HSP1_ANTLE        RYS-----RR GRRRY----- -
HSP1_ANTST        RYS-----RR GRRRY----- -
HSP1_MURLO        RYS-----RR GRRRY----- -
HSP1_MYRFA        RYS-----RR GRRRY----- -
HSP1_NINRI        RYS-----RR GRRRY----- -
HSP1_SMIGA        RYS-----RR GRRRY----- -
HSP1_SMIGI        RYS-----RR GRRRY----- -
HSP1_PHACL        RYS-----RR GRRRY----- -
HSP1_SMIMU        RYS-----RR GRRRY----- -
HSP1_SMIVI        RYS-----RR GRRRY----- -
HSP1_THYCY        RYS-----RR GRRRY----- -
HSP1_SMIAI        RYS-----RR GRRRY----- -
HSP1_SMICR        RYS-----RR GRRRY----- -
HSP1_SMIDL        RYS-----RR GRRRY----- -
HSP1_ANTLA        RYS-----RR GRRRY----- -
HSP1_PLAMS        RYS-----RR GRRRY----- -
HSP1_PLAIN        RCS-----RR RRRRC----- -
HSP1_PLAGI        RCS-----RR RRRRC----- -
HSP1_PLATE        RCS-----RR RRRRC----- -
```

Supplemental Figure 4. Alignment of Metatherian P1 type sperm protamines.
